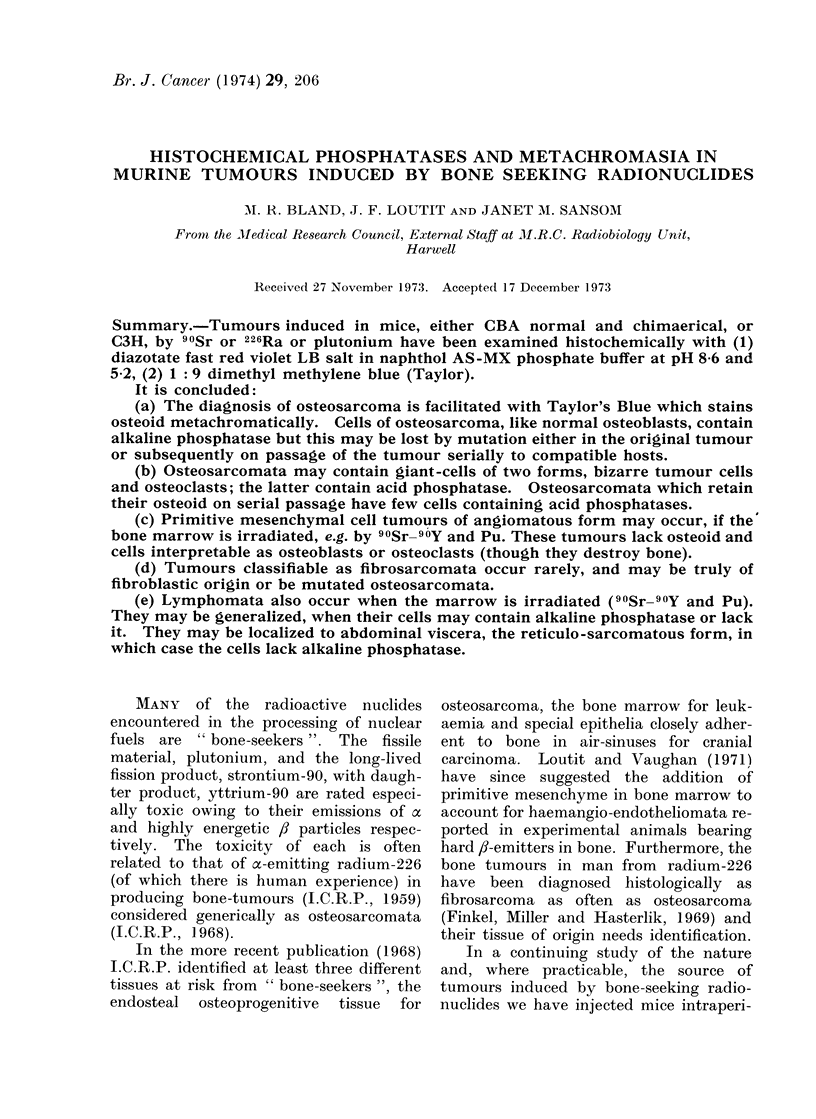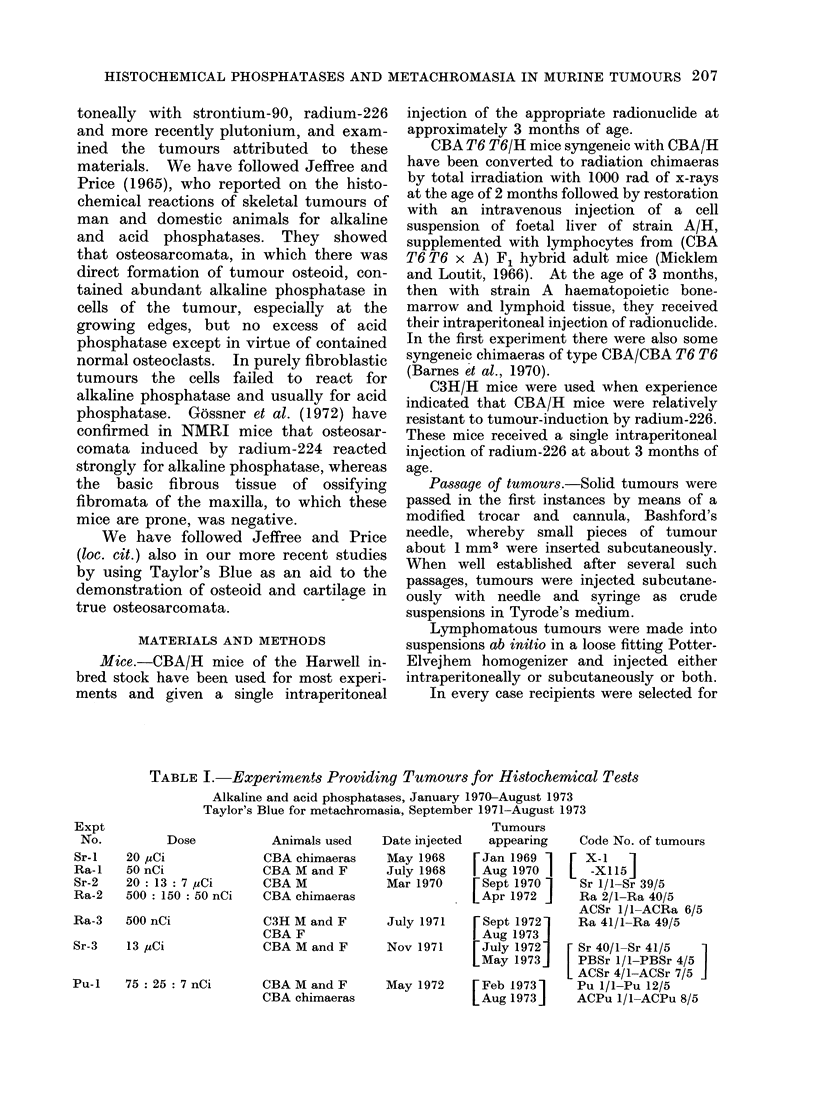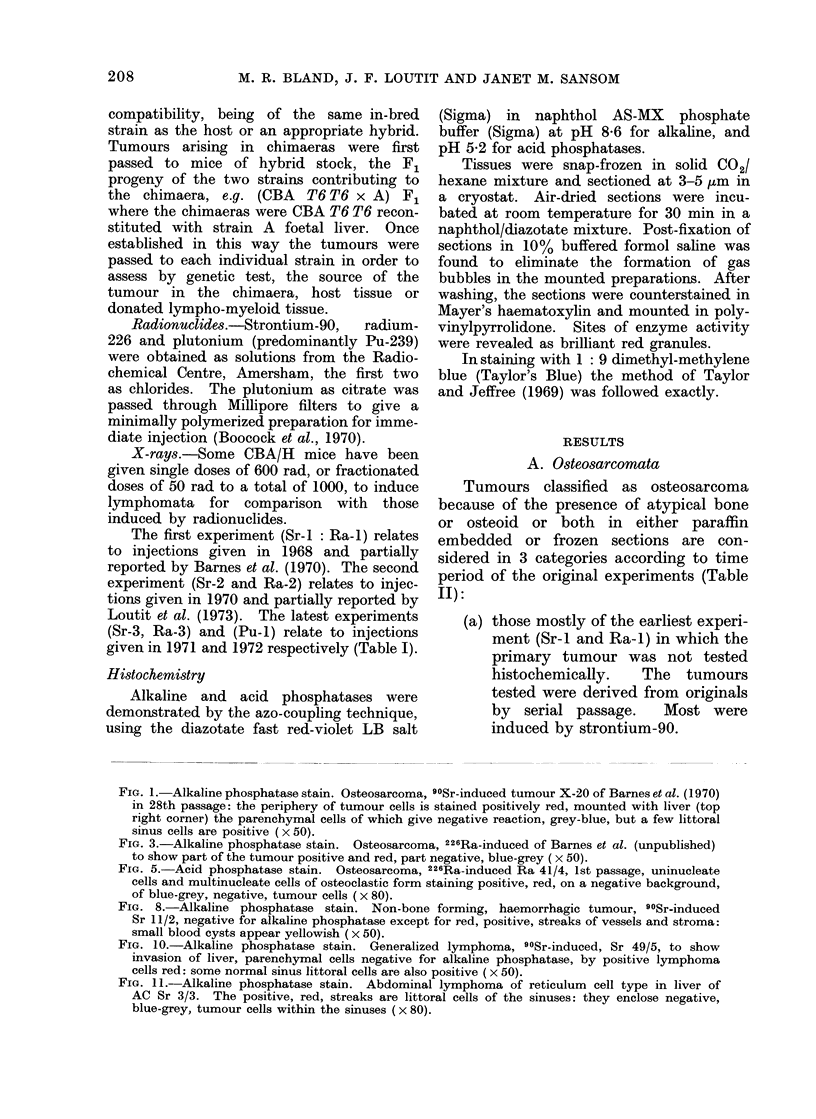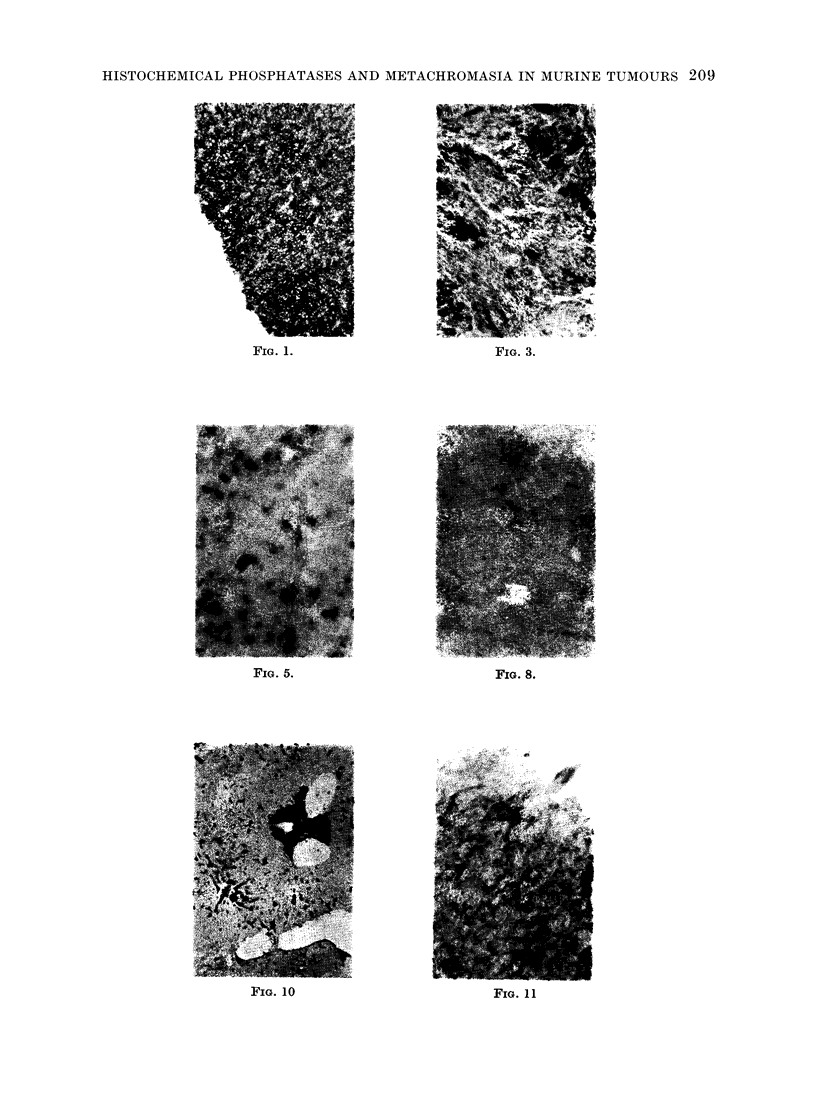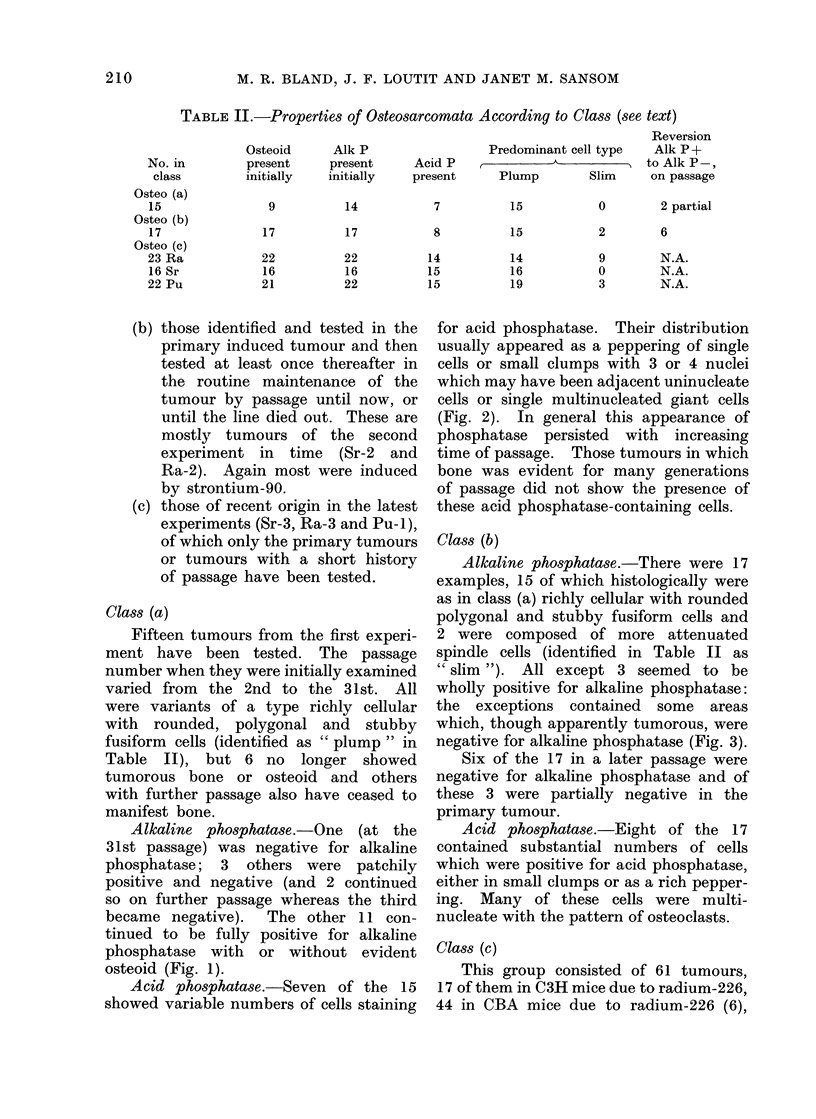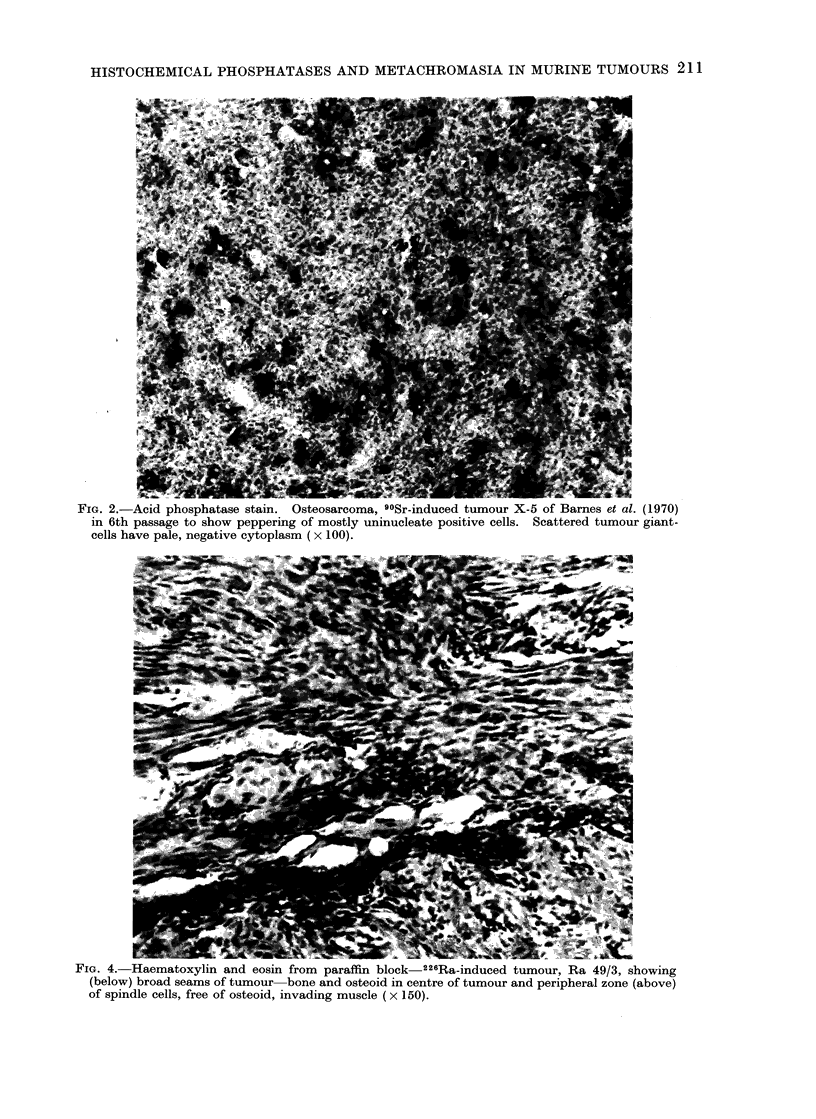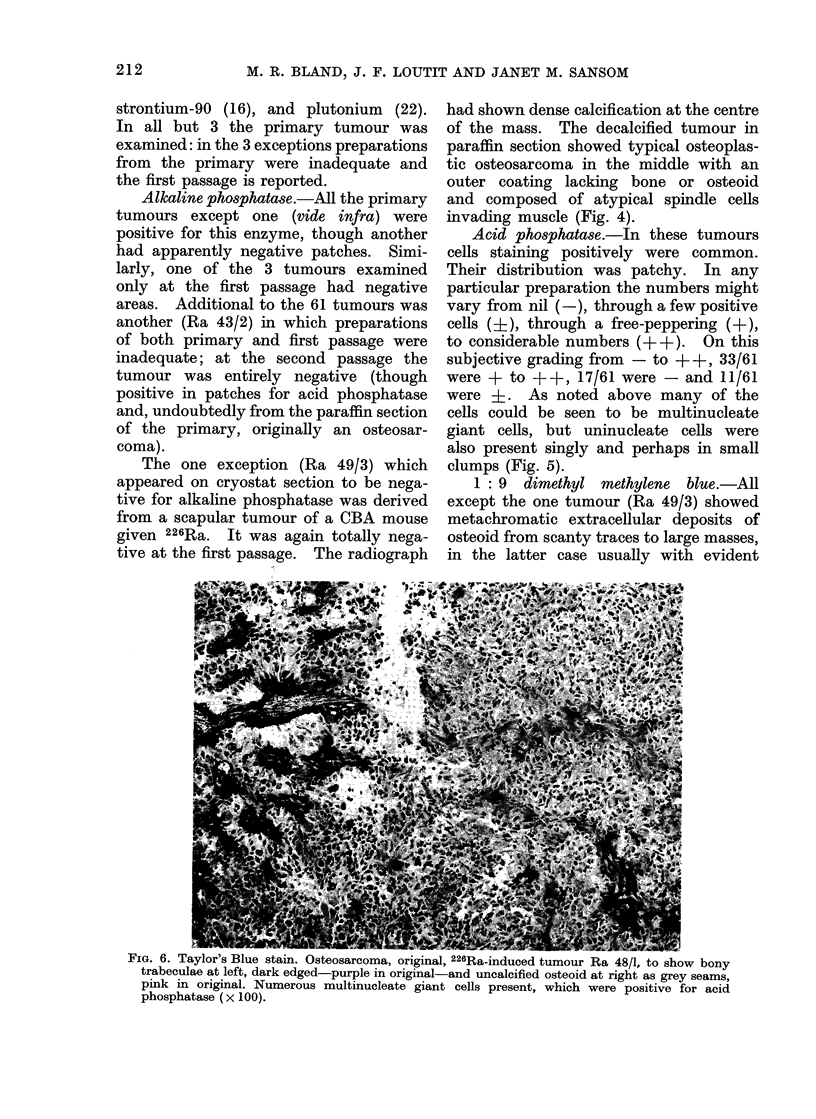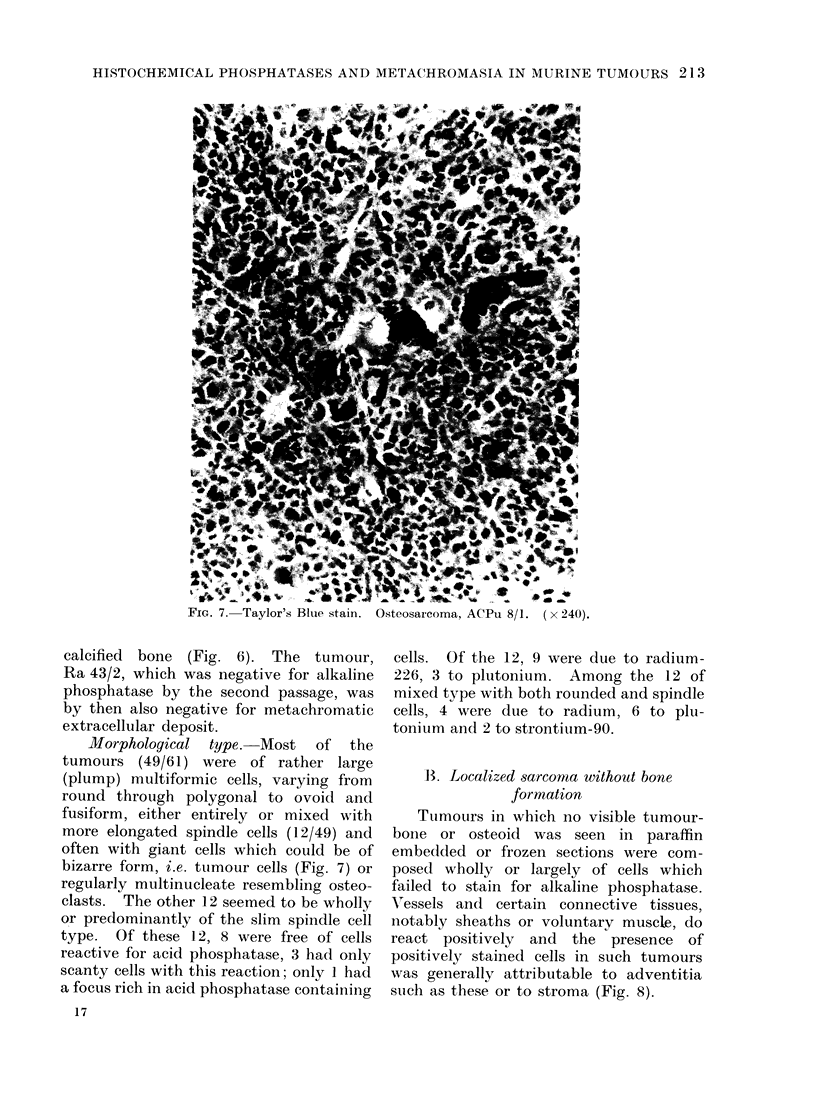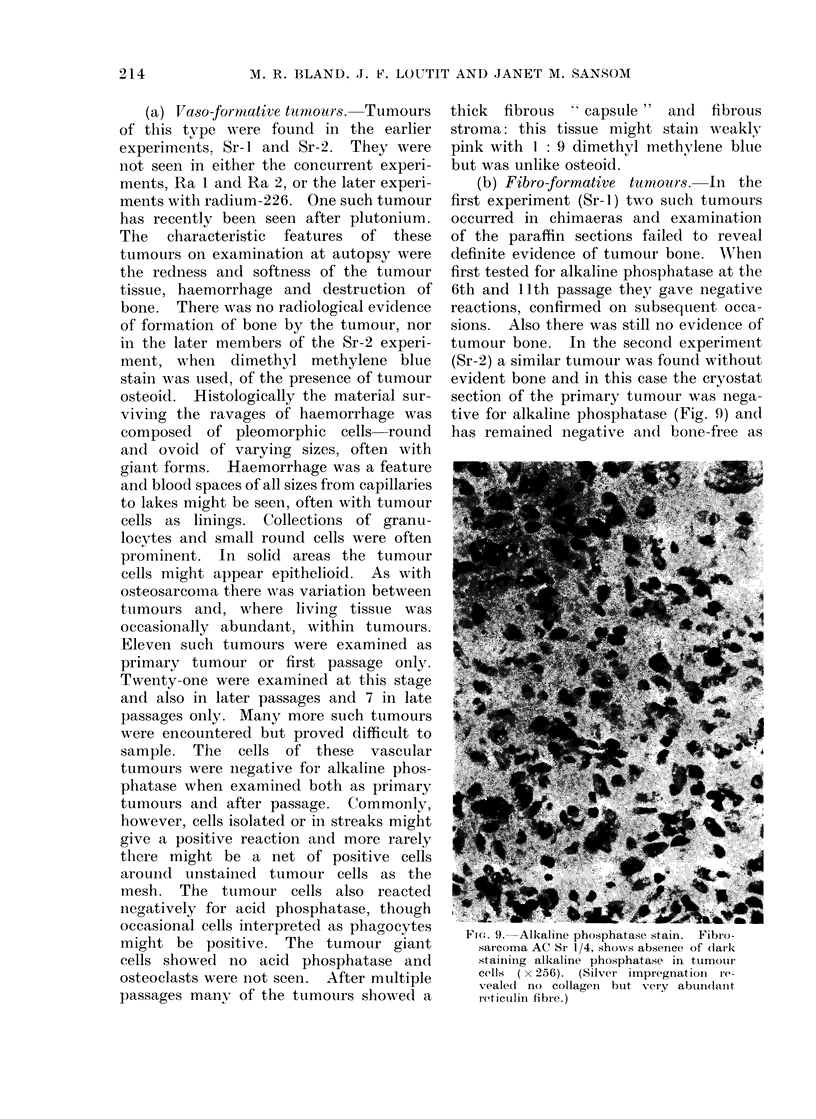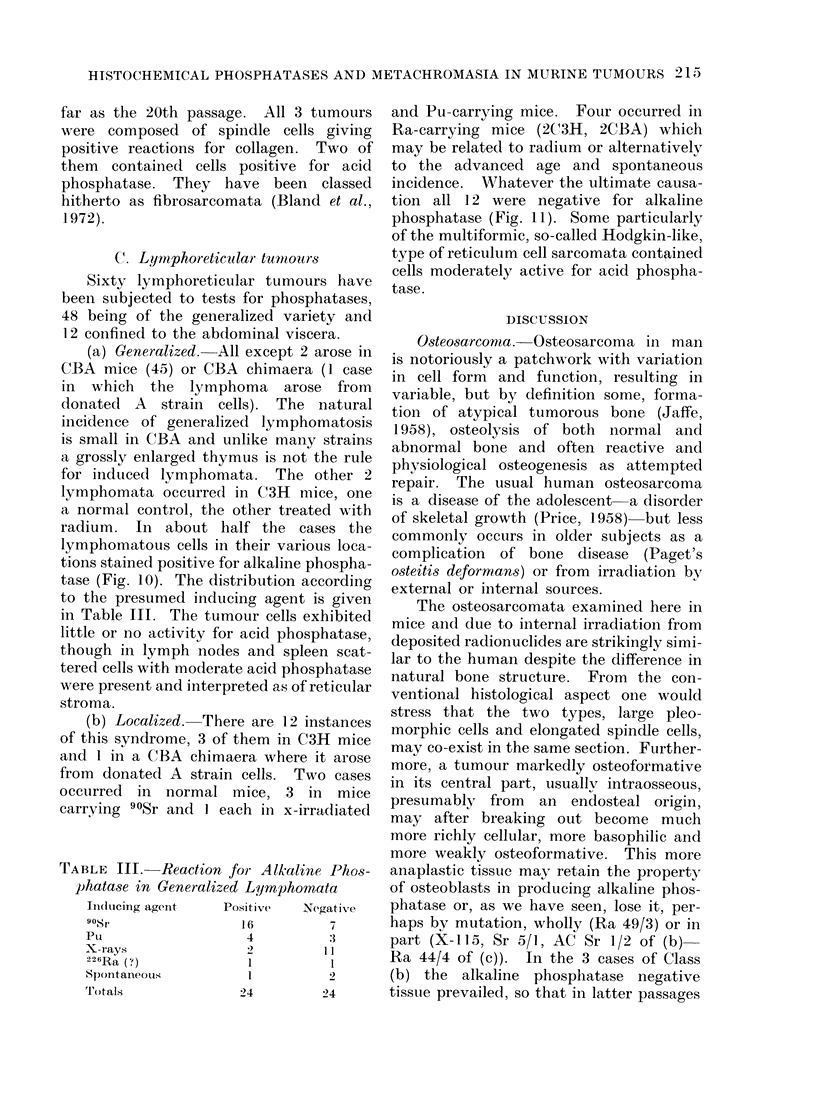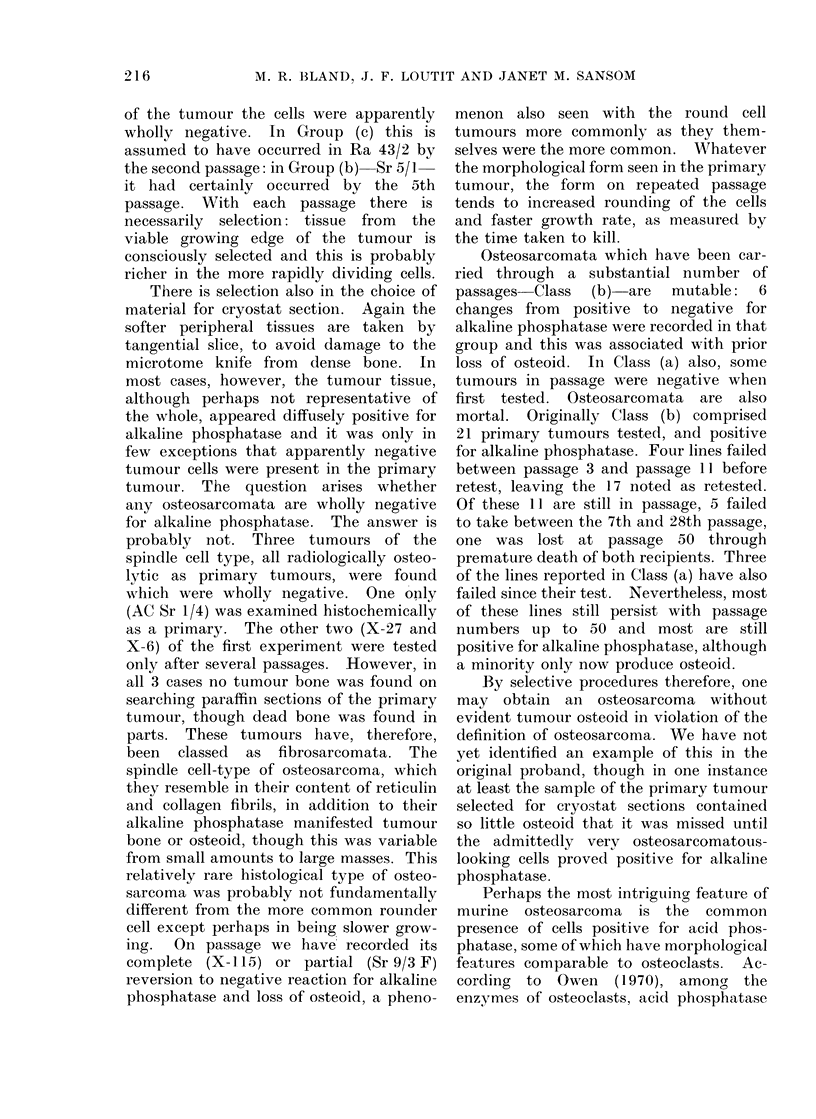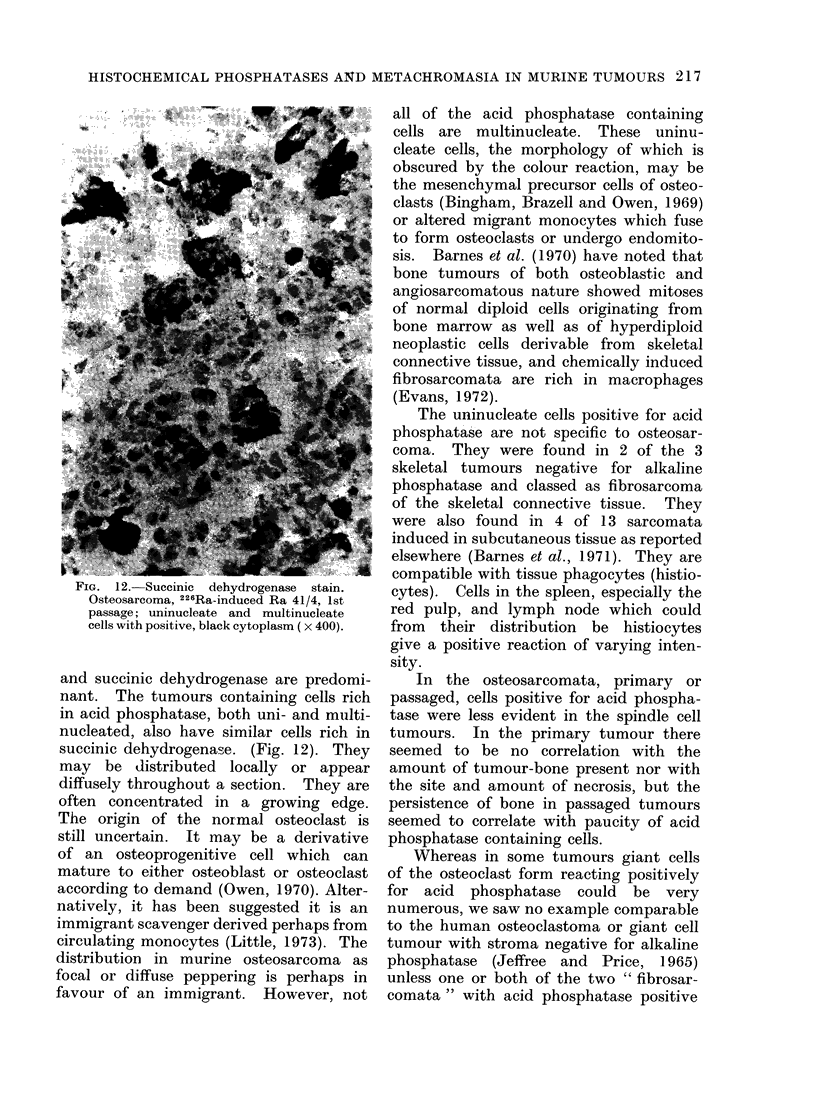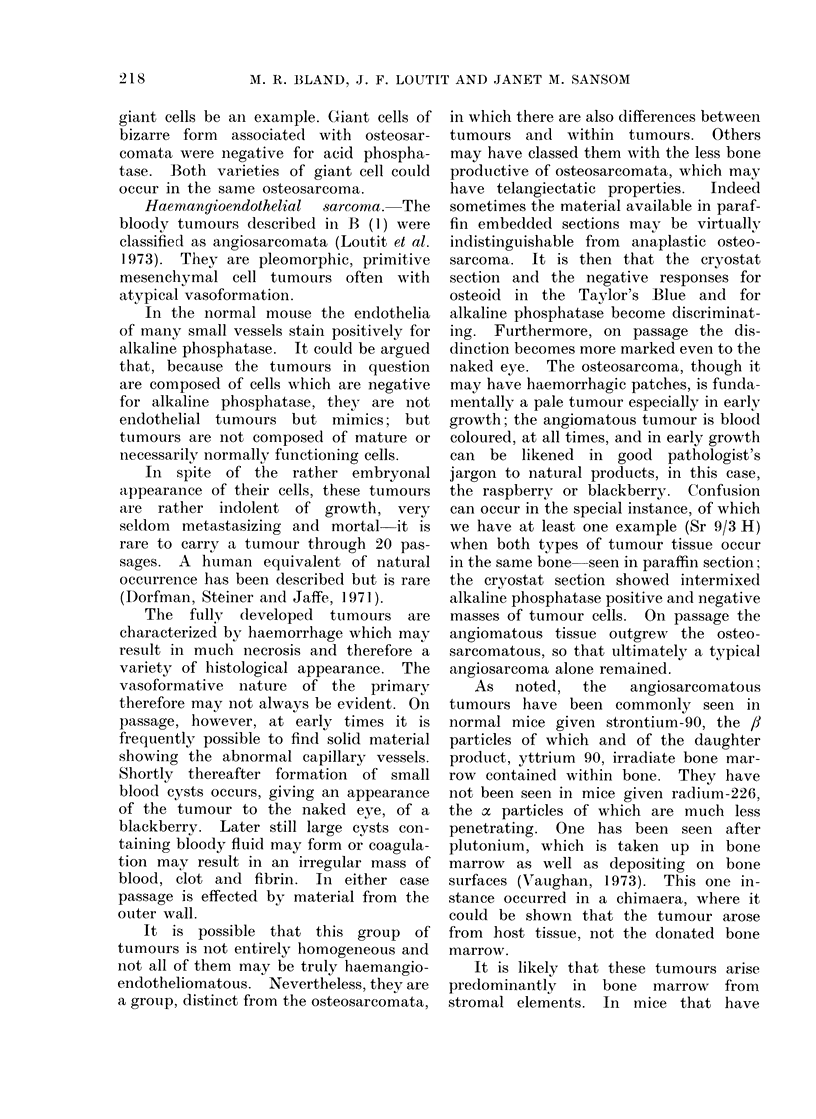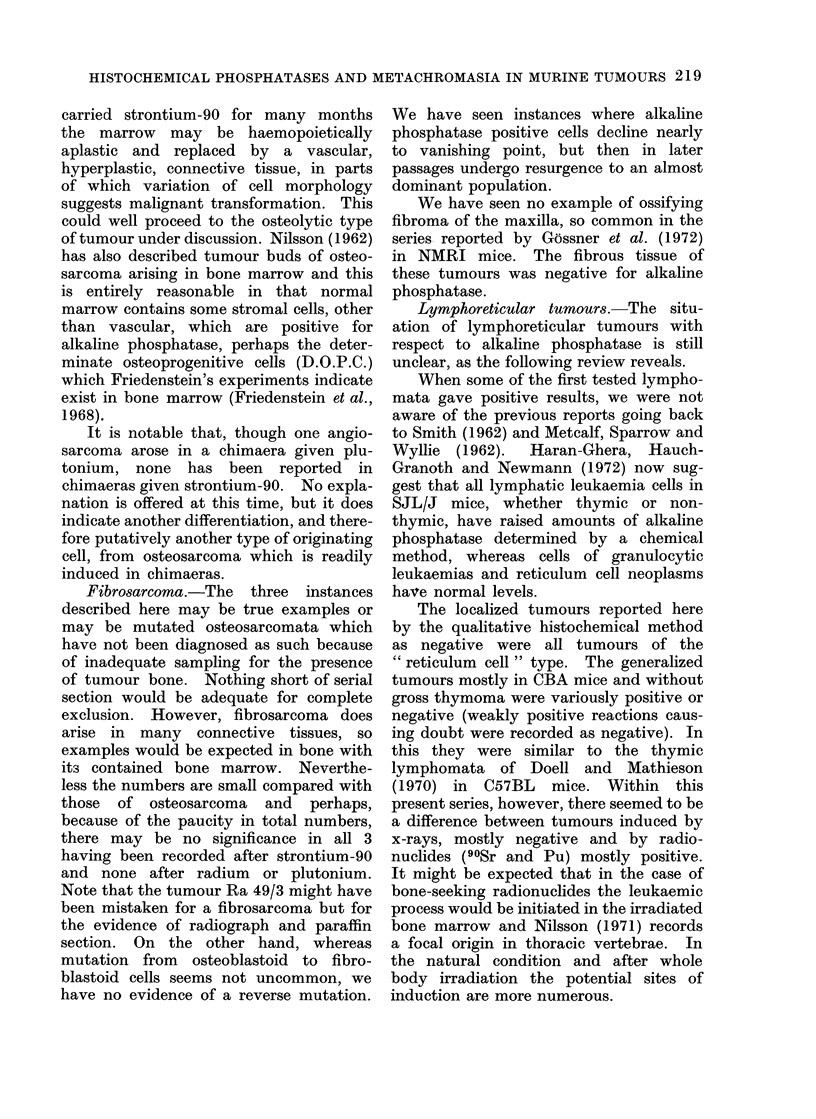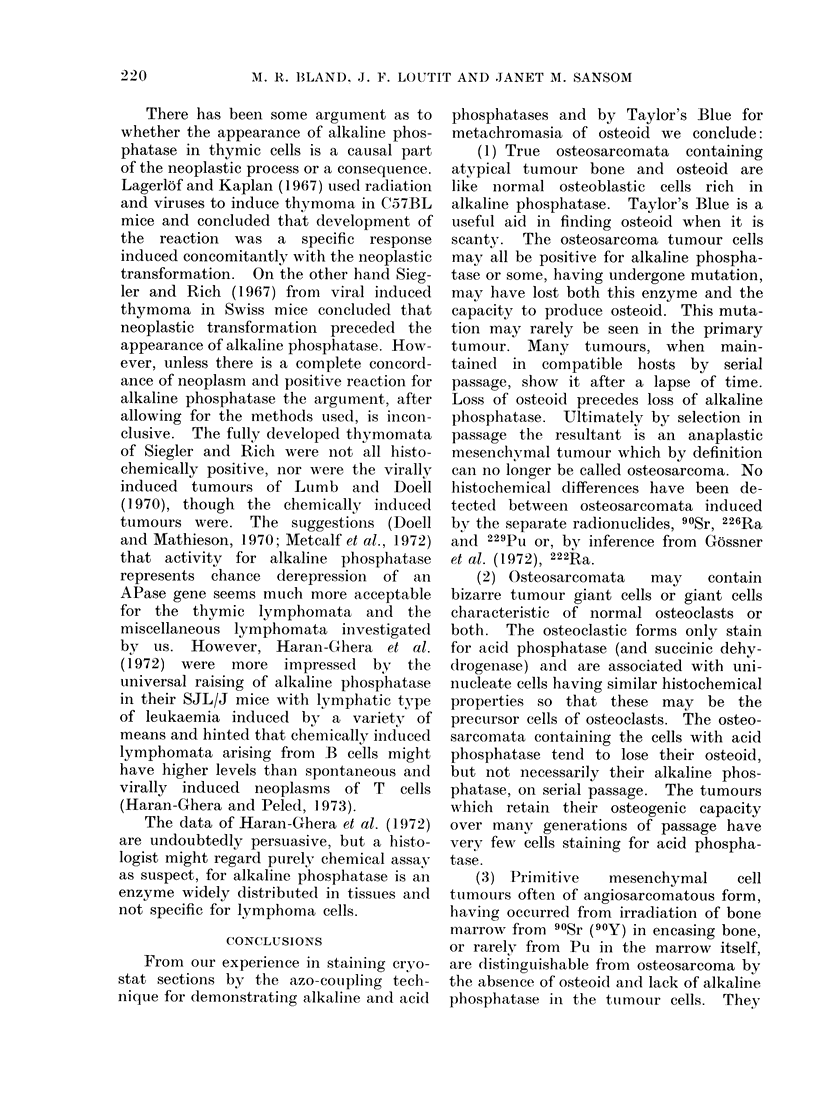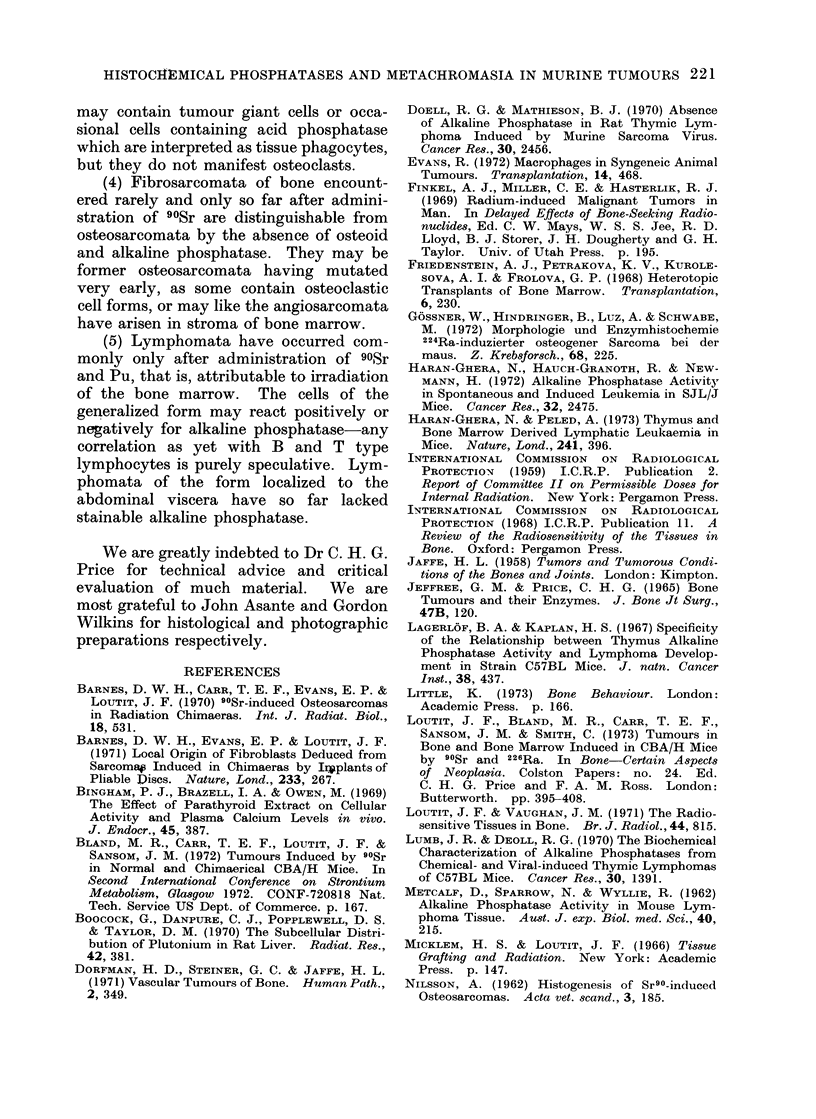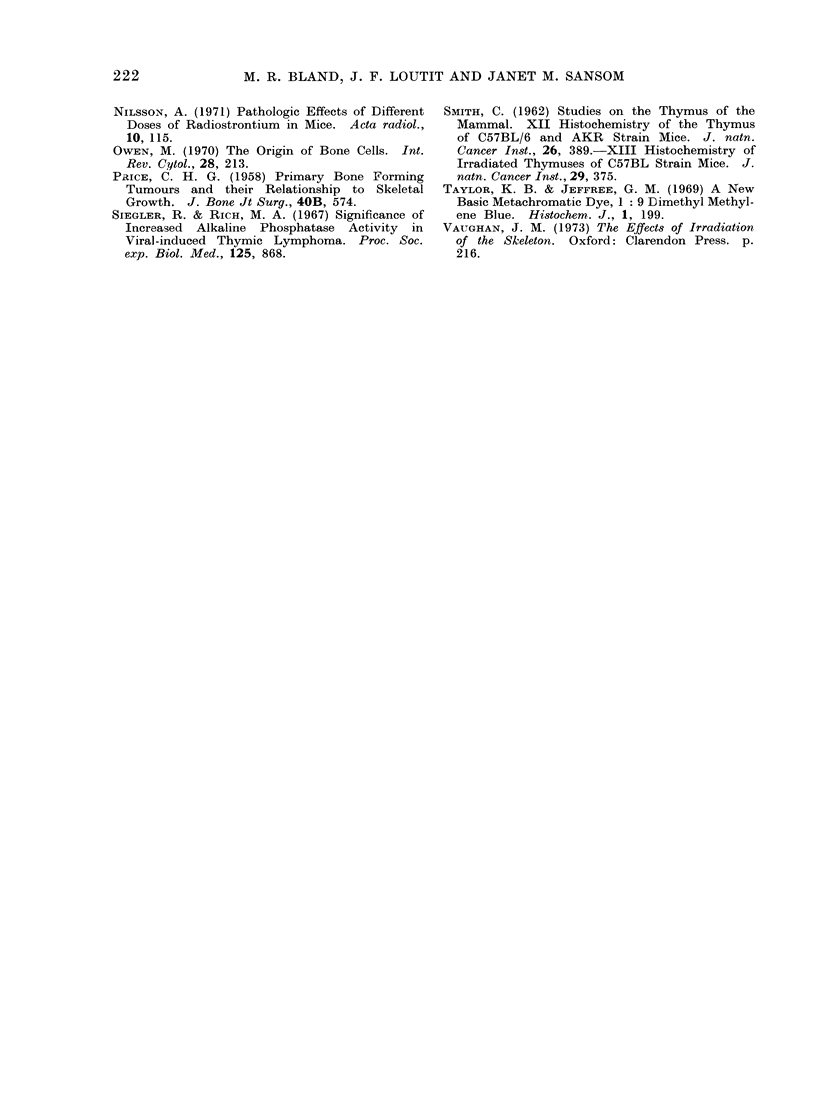# Histochemical Phosphatases and Metachromasia in Murine Tumours Induced by Bone Seeking Radionuclides

**DOI:** 10.1038/bjc.1974.60

**Published:** 1974-03

**Authors:** M. R. Bland, J. F. Loutit, Janet M. Sansom

## Abstract

**Images:**


Br. J. Cancer (1974) 29, 206

### HISTOCHEMICAL PHOSPHATASES AND METACHROMASIA IN MURINE TUMOURS INDUCED BY BONE SEEKING RADIONUCLIDES

M. R. BLAND, J. F. LOUTIT AND JANET M. SANSOM

*From the Medical Research Council, External Staff at M.R.C. Radiobiology Unit, Harwell*

Received 27 November 1973. Accepted 17 December 1973

**Summary.—Tumours induced in mice, either CBA normal and chimaerical, or C3H, by $^{90}$Sr or $^{226}$Ra or plutonium have been examined histochemically with (1) diazotate fast red violet LB salt in naphthol AS-MX phosphate buffer at pH 8·6 and 5·2, (2) 1 : 9 dimethyl methylene blue (Taylor).**

**It is concluded:**

**(a) The diagnosis of osteosarcoma is facilitated with Taylor's Blue which stains osteoid metachromatically. Cells of osteosarcoma, like normal osteoblasts, contain alkaline phosphatase but this may be lost by mutation either in the original tumour or subsequently on passage of the tumour serially to compatible hosts.**

**(b) Osteosarcomata may contain giant-cells of two forms, bizarre tumour cells and osteoclasts; the latter contain acid phosphatase. Osteosarcomata which retain their osteoid on serial passage have few cells containing acid phosphatases.**

**(c) Primitive mesenchymal cell tumours of angiomatous form may occur, if the bone marrow is irradiated, *e.g.* by $^{90}$Sr–$^{90}$Y and Pu. These tumours lack osteoid and cells interpretable as osteoblasts or osteoclasts (though they destroy bone).**

**(d) Tumours classifiable as fibrosarcomata occur rarely, and may be truly of fibroblastic origin or be mutated osteosarcomata.**

**(e) Lymphomata also occur when the marrow is irradiated ($^{90}$Sr–$^{90}$Y and Pu). They may be generalized, when their cells may contain alkaline phosphatase or lack it. They may be localized to abdominal viscera, the reticulo-sarcomatous form, in which case the cells lack alkaline phosphatase.**

MANY of the radioactive nuclides encountered in the processing of nuclear fuels are " bone-seekers ". The fissile material, plutonium, and the long-lived fission product, strontium-90, with daughter product, yttrium-90 are rated especially toxic owing to their emissions of $\alpha$ and highly energetic $\beta$ particles respectively. The toxicity of each is often related to that of $\alpha$-emitting radium-226 (of which there is human experience) in producing bone-tumours (I.C.R.P., 1959) considered generically as osteosarcomata (I.C.R.P., 1968).

In the more recent publication (1968) I.C.R.P. identified at least three different tissues at risk from " bone-seekers ", the endosteal osteoprogenitive tissue for osteosarcoma, the bone marrow for leukaemia and special epithelia closely adherent to bone in air-sinuses for cranial carcinoma. Loutit and Vaughan (1971) have since suggested the addition of primitive mesenchyme in bone marrow to account for haemangio-endotheliomata reported in experimental animals bearing hard $\beta$-emitters in bone. Furthermore, the bone tumours in man from radium-226 have been diagnosed histologically as fibrosarcoma as often as osteosarcoma (Finkel, Miller and Hasterlik, 1969) and their tissue of origin needs identification.

In a continuing study of the nature and, where practicable, the source of tumours induced by bone-seeking radionuclides we have injected mice intraperitoneally with strontium-90, radium-226 and more recently plutonium, and examined the tumours attributed to these materials. We have followed Jeffree and Price (1965), who reported on the histochemical reactions of skeletal tumours of man and domestic animals for alkaline and acid phosphatases. They showed that osteosarcomata, in which there was direct formation of tumour osteoid, contained abundant alkaline phosphatase in cells of the tumour, especially at the growing edges, but no excess of acid phosphatase except in virtue of contained normal osteoclasts. In purely fibroblastic tumours the cells failed to react for alkaline phosphatase and usually for acid phosphatase. Gössner *et al.* (1972) have confirmed in NMRI mice that osteosarcomata induced by radium-224 reacted strongly for alkaline phosphatase, whereas the basic fibrous tissue of ossifying fibromata of the maxilla, to which these mice are prone, was negative.

We have followed Jeffree and Price (*loc. cit.*) also in our more recent studies by using Taylor's Blue as an aid to the demonstration of osteoid and cartilage in true osteosarcomata.

### MATERIALS AND METHODS

*Mice.*—CBA/H mice of the Harwell inbred stock have been used for most experiments and given a single intraperitoneal injection of the appropriate radionuclide at approximately 3 months of age.

CBA *T6 T6*/H mice syngeneic with CBA/H have been converted to radiation chimaeras by total irradiation with 1000 rad of x-rays at the age of 2 months followed by restoration with an intravenous injection of a cell suspension of foetal liver of strain A/H, supplemented with lymphocytes from (CBA *T6 T6* × A) $F_1$ hybrid adult mice (Micklem and Loutit, 1966). At the age of 3 months, then with strain A haematopoietic bone-marrow and lymphoid tissue, they received their intraperitoneal injection of radionuclide. In the first experiment there were also some syngeneic chimaeras of type CBA/CBA *T6 T6* (Barnes *et al.*, 1970).

C3H/H mice were used when experience indicated that CBA/H mice were relatively resistant to tumour-induction by radium-226. These mice received a single intraperitoneal injection of radium-226 at about 3 months of age.

*Passage of tumours.*—Solid tumours were passed in the first instances by means of a modified trocar and cannula, Bashford's needle, whereby small pieces of tumour about 1 $mm^3$ were inserted subcutaneously. When well established after several such passages, tumours were injected subcutaneously with needle and syringe as crude suspensions in Tyrode's medium.

Lymphomatous tumours were made into suspensions *ab initio* in a loose fitting Potter-Elvejhem homogenizer and injected either intraperitoneally or subcutaneously or both.

In every case recipients were selected for

TABLE I.—*Experiments Providing Tumours for Histochemical Tests*

Alkaline and acid phosphatases, January 1970–August 1973
Taylor's Blue for metachromasia, September 1971–August 1973

| Expt No. | Dose | Animals used | Date injected | Tumours appearing | Code No. of tumours |
|---|---|---|---|---|---|
| Sr-1 | 20 μCi | CBA chimaeras | May 1968 | Jan 1969 | X-1 |
| Ra-1 | 50 nCi | CBA M and F | July 1968 | Aug 1970 | -X115 |
| Sr-2 | 20 : 13 : 7 μCi | CBA M | Mar 1970 | Sept 1970 | Sr 1/1–Sr 39/5 |
| Ra-2 | 500 : 150 : 50 nCi | CBA chimaeras | | Apr 1972 | Ra 2/1–Ra 40/5 |
| | | | | | ACSr 1/1–ACRa 6/5 |
| Ra-3 | 500 nCi | C3H M and F | July 1971 | Sept 1972 | Ra 41/1–Ra 49/5 |
| | | CBA F | | Aug 1973 | |
| Sr-3 | 13 μCi | CBA M and F | Nov 1971 | July 1972 | Sr 40/1–Sr 41/5 |
| | | | | May 1973 | PBSr 1/1–PBSr 4/5 |
| | | | | | ACSr 4/1–ACSr 7/5 |
| Pu-1 | 75 : 25 : 7 nCi | CBA M and F | May 1972 | Feb 1973 | Pu 1/1–Pu 12/5 |
| | | CBA chimaeras | | Aug 1973 | ACPu 1/1–ACPu 8/5 |

compatibility, being of the same in-bred strain as the host or an appropriate hybrid. Tumours arising in chimaeras were first passed to mice of hybrid stock, the $F_1$ progeny of the two strains contributing to the chimaera, *e.g.* (CBA *T6 T6* × A) $F_1$ where the chimaeras were CBA *T6 T6* reconstituted with strain A foetal liver. Once established in this way the tumours were passed to each individual strain in order to assess by genetic test, the source of the tumour in the chimaera, host tissue or donated lympho-myeloid tissue.

*Radionuclides.*—Strontium-90, radium-226 and plutonium (predominantly Pu-239) were obtained as solutions from the Radiochemical Centre, Amersham, the first two as chlorides. The plutonium as citrate was passed through Millipore filters to give a minimally polymerized preparation for immediate injection (Boocock *et al.*, 1970).

*X-rays.*—Some CBA/H mice have been given single doses of 600 rad, or fractionated doses of 50 rad to a total of 1000, to induce lymphomata for comparison with those induced by radionuclides.

The first experiment (Sr-1 : Ra-1) relates to injections given in 1968 and partially reported by Barnes *et al.* (1970). The second experiment (Sr-2 and Ra-2) relates to injections given in 1970 and partially reported by Loutit *et al.* (1973). The latest experiments (Sr-3, Ra-3) and (Pu-1) relate to injections given in 1971 and 1972 respectively (Table I).

*Histochemistry*

Alkaline and acid phosphatases were demonstrated by the azo-coupling technique, using the diazotate fast red-violet LB salt (Sigma) in naphthol AS-MX phosphate buffer (Sigma) at pH 8·6 for alkaline, and pH 5·2 for acid phosphatases.

Tissues were snap-frozen in solid $CO_2$/hexane mixture and sectioned at 3–5 $\mu$m in a cryostat. Air-dried sections were incubated at room temperature for 30 min in a naphthol/diazotate mixture. Post-fixation of sections in 10% buffered formol saline was found to eliminate the formation of gas bubbles in the mounted preparations. After washing, the sections were counterstained in Mayer's haematoxylin and mounted in polyvinylpyrrolidone. Sites of enzyme activity were revealed as brilliant red granules.

In staining with 1 : 9 dimethyl-methylene blue (Taylor's Blue) the method of Taylor and Jeffree (1969) was followed exactly.

### RESULTS

### A. *Osteosarcomata*

Tumours classified as osteosarcoma because of the presence of atypical bone or osteoid or both in either paraffin embedded or frozen sections are considered in 3 categories according to time period of the original experiments (Table II):

(a) those mostly of the earliest experiment (Sr-1 and Ra-1) in which the primary tumour was not tested histochemically. The tumours tested were derived from originals by serial passage. Most were induced by strontium-90.

FIG. 1.—Alkaline phosphatase stain. Osteosarcoma, $^{90}$Sr-induced tumour X-20 of Barnes *et al.* (1970) in 28th passage: the periphery of tumour cells is stained positively red, mounted with liver (top right corner) the parenchymal cells of which give negative reaction, grey-blue, but a few littoral sinus cells are positive (× 50).

FIG. 3.—Alkaline phosphatase stain. Osteosarcoma, $^{226}$Ra-induced of Barnes *et al.* (unpublished) to show part of the tumour positive and red, part negative, blue-grey (× 50).

FIG. 5.—Acid phosphatase stain. Osteosarcoma, $^{226}$Ra-induced Ra 41/4, 1st passage, uninucleate cells and multinucleate cells of osteoclastic form staining positive, red, on a negative background, of blue-grey, negative, tumour cells (× 80).

FIG. 8.—Alkaline phosphatase stain. Non-bone forming, haemorrhagic tumour, $^{90}$Sr-induced Sr 11/2, negative for alkaline phosphatase except for red, positive, streaks of vessels and stroma: small blood cysts appear yellowish (× 50).

FIG. 10.—Alkaline phosphatase stain. Generalized lymphoma, $^{90}$Sr-induced, Sr 49/5, to show invasion of liver, parenchymal cells negative for alkaline phosphatase, by positive lymphoma cells red: some normal sinus littoral cells are also positive (× 50).

FIG. 11.—Alkaline phosphatase stain. Abdominal lymphoma of reticulum cell type in liver of AC Sr 3/3. The positive, red, streaks are littoral cells of the sinuses: they enclose negative, blue-grey, tumour cells within the sinuses (× 80).

FIG. 1.

FIG. 3.

FIG. 5.

FIG. 8.

FIG. 10

FIG. 11

TABLE II.—*Properties of Osteosarcomata According to Class (see text)*

| No. in class | Osteoid present initially | Alk P present initially | Acid P present | Predominant cell type | | Reversion Alk P+ to Alk P−, on passage |
|---|---|---|---|---|---|---|
| | | | | Plump | Slim | |
| Osteo (a) | | | | | | |
| 15 | 9 | 14 | 7 | 15 | 0 | 2 partial |
| Osteo (b) | | | | | | |
| 17 | 17 | 17 | 8 | 15 | 2 | 6 |
| Osteo (c) | | | | | | |
| 23 Ra | 22 | 22 | 14 | 14 | 9 | N.A. |
| 16 Sr | 16 | 16 | 15 | 16 | 0 | N.A. |
| 22 Pu | 21 | 22 | 15 | 19 | 3 | N.A. |

(b) those identified and tested in the primary induced tumour and then tested at least once thereafter in the routine maintenance of the tumour by passage until now, or until the line died out. These are mostly tumours of the second experiment in time (Sr-2 and Ra-2). Again most were induced by strontium-90.

(c) those of recent origin in the latest experiments (Sr-3, Ra-3 and Pu-1), of which only the primary tumours or tumours with a short history of passage have been tested.

*Class (a)*

Fifteen tumours from the first experiment have been tested. The passage number when they were initially examined varied from the 2nd to the 31st. All were variants of a type richly cellular with rounded, polygonal and stubby fusiform cells (identified as " plump " in Table II), but 6 no longer showed tumorous bone or osteoid and others with further passage also have ceased to manifest bone.

*Alkaline phosphatase.*—One (at the 31st passage) was negative for alkaline phosphatase; 3 others were patchily positive and negative (and 2 continued so on further passage whereas the third became negative). The other 11 continued to be fully positive for alkaline phosphatase with or without evident osteoid (Fig. 1).

*Acid phosphatase.*—Seven of the 15 showed variable numbers of cells staining for acid phosphatase. Their distribution usually appeared as a peppering of single cells or small clumps with 3 or 4 nuclei which may have been adjacent uninucleate cells or single multinucleated giant cells (Fig. 2). In general this appearance of phosphatase persisted with increasing time of passage. Those tumours in which bone was evident for many generations of passage did not show the presence of these acid phosphatase-containing cells.

*Class (b)*

*Alkaline phosphatase.*—There were 17 examples, 15 of which histologically were as in class (a) richly cellular with rounded polygonal and stubby fusiform cells and 2 were composed of more attenuated spindle cells (identified in Table II as " slim "). All except 3 seemed to be wholly positive for alkaline phosphatase: the exceptions contained some areas which, though apparently tumorous, were negative for alkaline phosphatase (Fig. 3).

Six of the 17 in a later passage were negative for alkaline phosphatase and of these 3 were partially negative in the primary tumour.

*Acid phosphatase.*—Eight of the 17 contained substantial numbers of cells which were positive for acid phosphatase, either in small clumps or as a rich peppering. Many of these cells were multinucleate with the pattern of osteoclasts.

*Class (c)*

This group consisted of 61 tumours, 17 of them in C3H mice due to radium-226, 44 in CBA mice due to radium-226 (6),

FIG. 2.—Acid phosphatase stain. Osteosarcoma, $^{90}Sr$-induced tumour X-5 of Barnes *et al.* (1970) in 6th passage to show peppering of mostly uninucleate positive cells. Scattered tumour giant-cells have pale, negative cytoplasm (× 100).

FIG. 4.—Haematoxylin and eosin from paraffin block—$^{226}Ra$-induced tumour, Ra 49/3, showing (below) broad seams of tumour—bone and osteoid in centre of tumour and peripheral zone (above) of spindle cells, free of osteoid, invading muscle (× 150).

strontium-90 (16), and plutonium (22). In all but 3 the primary tumour was examined: in the 3 exceptions preparations from the primary were inadequate and the first passage is reported.

*Alkaline phosphatase.*—All the primary tumours except one (*vide infra*) were positive for this enzyme, though another had apparently negative patches. Similarly, one of the 3 tumours examined only at the first passage had negative areas. Additional to the 61 tumours was another (Ra 43/2) in which preparations of both primary and first passage were inadequate; at the second passage the tumour was entirely negative (though positive in patches for acid phosphatase and, undoubtedly from the paraffin section of the primary, originally an osteosarcoma).

The one exception (Ra 49/3) which appeared on cryostat section to be negative for alkaline phosphatase was derived from a scapular tumour of a CBA mouse given $^{226}$Ra. It was again totally negative at the first passage. The radiograph had shown dense calcification at the centre of the mass. The decalcified tumour in paraffin section showed typical osteoplastic osteosarcoma in the middle with an outer coating lacking bone or osteoid and composed of atypical spindle cells invading muscle (Fig. 4).

*Acid phosphatase.*—In these tumours cells staining positively were common. Their distribution was patchy. In any particular preparation the numbers might vary from nil (—), through a few positive cells (±), through a free-peppering (+), to considerable numbers (++). On this subjective grading from — to ++, 33/61 were + to ++, 17/61 were — and 11/61 were ±. As noted above many of the cells could be seen to be multinucleate giant cells, but uninucleate cells were also present singly and perhaps in small clumps (Fig. 5).

1 : 9 *dimethyl methylene blue.*—All except the one tumour (Ra 49/3) showed metachromatic extracellular deposits of osteoid from scanty traces to large masses, in the latter case usually with evident

FIG. 6. Taylor's Blue stain. Osteosarcoma, original, $^{226}$Ra-induced tumour Ra 48/1, to show bony trabeculae at left, dark edged—purple in original—and uncalcified osteoid at right as grey seams, pink in original. Numerous multinucleate giant cells present, which were positive for acid phosphatase (× 100).

FIG. 7.—Taylor's Blue stain. Osteosarcoma, ACPu 8/1. (×240).

calcified bone (Fig. 6). The tumour, Ra 43/2, which was negative for alkaline phosphatase by the second passage, was by then also negative for metachromatic extracellular deposit.

*Morphological type*.—Most of the tumours (49/61) were of rather large (plump) multiformic cells, varying from round through polygonal to ovoid and fusiform, either entirely or mixed with more elongated spindle cells (12/49) and often with giant cells which could be of bizarre form, *i.e.* tumour cells (Fig. 7) or regularly multinucleate resembling osteoclasts. The other 12 seemed to be wholly or predominantly of the slim spindle cell type. Of these 12, 8 were free of cells reactive for acid phosphatase, 3 had only scanty cells with this reaction; only 1 had a focus rich in acid phosphatase containing cells. Of the 12, 9 were due to radium-226, 3 to plutonium. Among the 12 of mixed type with both rounded and spindle cells, 4 were due to radium, 6 to plutonium and 2 to strontium-90.

### B. *Localized sarcoma without bone formation*

Tumours in which no visible tumour-bone or osteoid was seen in paraffin embedded or frozen sections were composed wholly or largely of cells which failed to stain for alkaline phosphatase. Vessels and certain connective tissues, notably sheaths or voluntary muscle, do react positively and the presence of positively stained cells in such tumours was generally attributable to adventitia such as these or to stroma (Fig. 8).

(a) *Vaso-formative tumours.*—Tumours of this type were found in the earlier experiments, Sr-1 and Sr-2. They were not seen in either the concurrent experiments, Ra 1 and Ra 2, or the later experiments with radium-226. One such tumour has recently been seen after plutonium. The characteristic features of these tumours on examination at autopsy were the redness and softness of the tumour tissue, haemorrhage and destruction of bone. There was no radiological evidence of formation of bone by the tumour, nor in the later members of the Sr-2 experiment, when dimethyl methylene blue stain was used, of the presence of tumour osteoid. Histologically the material surviving the ravages of haemorrhage was composed of pleomorphic cells—round and ovoid of varying sizes, often with giant forms. Haemorrhage was a feature and blood spaces of all sizes from capillaries to lakes might be seen, often with tumour cells as linings. Collections of granulocytes and small round cells were often prominent. In solid areas the tumour cells might appear epithelioid. As with osteosarcoma there was variation between tumours and, where living tissue was occasionally abundant, within tumours. Eleven such tumours were examined as primary tumour or first passage only. Twenty-one were examined at this stage and also in later passages and 7 in late passages only. Many more such tumours were encountered but proved difficult to sample. The cells of these vascular tumours were negative for alkaline phosphatase when examined both as primary tumours and after passage. Commonly, however, cells isolated or in streaks might give a positive reaction and more rarely there might be a net of positive cells around unstained tumour cells as the mesh. The tumour cells also reacted negatively for acid phosphatase, though occasional cells interpreted as phagocytes might be positive. The tumour giant cells showed no acid phosphatase and osteoclasts were not seen. After multiple passages many of the tumours showed a thick fibrous "capsule" and fibrous stroma: this tissue might stain weakly pink with 1 : 9 dimethyl methylene blue but was unlike osteoid.

(b) *Fibro-formative tumours.*—In the first experiment (Sr-1) two such tumours occurred in chimaeras and examination of the paraffin sections failed to reveal definite evidence of tumour bone. When first tested for alkaline phosphatase at the 6th and 11th passage they gave negative reactions, confirmed on subsequent occasions. Also there was still no evidence of tumour bone. In the second experiment (Sr-2) a similar tumour was found without evident bone and in this case the cryostat section of the primary tumour was negative for alkaline phosphatase (Fig. 9) and has remained negative and bone-free as

FIG. 9.—Alkaline phosphatase stain. Fibrosarcoma AC Sr 1/4, shows absence of dark staining alkaline phosphatase in tumour cells ($\times 256$). (Silver impregnation revealed no collagen but very abundant reticulin fibre.)

far as the 20th passage. All 3 tumours were composed of spindle cells giving positive reactions for collagen. Two of them contained cells positive for acid phosphatase. They have been classed hitherto as fibrosarcomata (Bland *et al.*, 1972).

### C. *Lymphoreticular tumours*

Sixty lymphoreticular tumours have been subjected to tests for phosphatases, 48 being of the generalized variety and 12 confined to the abdominal viscera.

(a) *Generalized*.—All except 2 arose in CBA mice (45) or CBA chimaera (1 case in which the lymphoma arose from donated A strain cells). The natural incidence of generalized lymphomatosis is small in CBA and unlike many strains a grossly enlarged thymus is not the rule for induced lymphomata. The other 2 lymphomata occurred in C3H mice, one a normal control, the other treated with radium. In about half the cases the lymphomatous cells in their various locations stained positive for alkaline phosphatase (Fig. 10). The distribution according to the presumed inducing agent is given in Table III. The tumour cells exhibited little or no activity for acid phosphatase, though in lymph nodes and spleen scattered cells with moderate acid phosphatase were present and interpreted as of reticular stroma.

(b) *Localized*.—There are 12 instances of this syndrome, 3 of them in C3H mice and 1 in a CBA chimaera where it arose from donated A strain cells. Two cases occurred in normal mice, 3 in mice carrying $^{90}$Sr and 1 each in x-irradiated and Pu-carrying mice. Four occurred in Ra-carrying mice (2C3H, 2CBA) which may be related to radium or alternatively to the advanced age and spontaneous incidence. Whatever the ultimate causation all 12 were negative for alkaline phosphatase (Fig. 11). Some particularly of the multiformic, so-called Hodgkin-like, type of reticulum cell sarcomata contained cells moderately active for acid phosphatase.

TABLE III.—*Reaction for Alkaline Phosphatase in Generalized Lymphomata*

| Inducing agent | Positive | Negative |
|---|---|---|
| $^{90}$Sr | 16 | 7 |
| Pu | 4 | 3 |
| X-rays | 2 | 11 |
| $^{226}$Ra (?) | 1 | 1 |
| Spontaneous | 1 | 2 |
| Totals | 24 | 24 |

### DISCUSSION

*Osteosarcoma*.—Osteosarcoma in man is notoriously a patchwork with variation in cell form and function, resulting in variable, but by definition some, formation of atypical tumorous bone (Jaffe, 1958), osteolysis of both normal and abnormal bone and often reactive and physiological osteogenesis as attempted repair. The usual human osteosarcoma is a disease of the adolescent—a disorder of skeletal growth (Price, 1958)—but less commonly occurs in older subjects as a complication of bone disease (Paget's *osteitis deformans*) or from irradiation by external or internal sources.

The osteosarcomata examined here in mice and due to internal irradiation from deposited radionuclides are strikingly similar to the human despite the difference in natural bone structure. From the conventional histological aspect one would stress that the two types, large pleomorphic cells and elongated spindle cells, may co-exist in the same section. Furthermore, a tumour markedly osteoformative in its central part, usually intraosseous, presumably from an endosteal origin, may after breaking out become much more richly cellular, more basophilic and more weakly osteoformative. This more anaplastic tissue may retain the property of osteoblasts in producing alkaline phosphatase or, as we have seen, lose it, perhaps by mutation, wholly (Ra 49/3) or in part (X-115, Sr 5/1, AC Sr 1/2 of (b)—Ra 44/4 of (c)). In the 3 cases of Class (b) the alkaline phosphatase negative tissue prevailed, so that in latter passages of the tumour the cells were apparently wholly negative. In Group (c) this is assumed to have occurred in Ra 43/2 by the second passage: in Group (b)—Sr 5/1—it had certainly occurred by the 5th passage. With each passage there is necessarily selection: tissue from the viable growing edge of the tumour is consciously selected and this is probably richer in the more rapidly dividing cells.

There is selection also in the choice of material for cryostat section. Again the softer peripheral tissues are taken by tangential slice, to avoid damage to the microtome knife from dense bone. In most cases, however, the tumour tissue, although perhaps not representative of the whole, appeared diffusely positive for alkaline phosphatase and it was only in few exceptions that apparently negative tumour cells were present in the primary tumour. The question arises whether any osteosarcomata are wholly negative for alkaline phosphatase. The answer is probably not. Three tumours of the spindle cell type, all radiologically osteolytic as primary tumours, were found which were wholly negative. One only (AC Sr 1/4) was examined histochemically as a primary. The other two (X-27 and X-6) of the first experiment were tested only after several passages. However, in all 3 cases no tumour bone was found on searching paraffin sections of the primary tumour, though dead bone was found in parts. These tumours have, therefore, been classed as fibrosarcomata. The spindle cell-type of osteosarcoma, which they resemble in their content of reticulin and collagen fibrils, in addition to their alkaline phosphatase manifested tumour bone or osteoid, though this was variable from small amounts to large masses. This relatively rare histological type of osteosarcoma was probably not fundamentally different from the more common rounder cell except perhaps in being slower growing. On passage we have recorded its complete (X-115) or partial (Sr 9/3 F) reversion to negative reaction for alkaline phosphatase and loss of osteoid, a phenomenon also seen with the round cell tumours more commonly as they themselves were the more common. Whatever the morphological form seen in the primary tumour, the form on repeated passage tends to increased rounding of the cells and faster growth rate, as measured by the time taken to kill.

Osteosarcomata which have been carried through a substantial number of passages—Class (b)—are mutable: 6 changes from positive to negative for alkaline phosphatase were recorded in that group and this was associated with prior loss of osteoid. In Class (a) also, some tumours in passage were negative when first tested. Osteosarcomata are also mortal. Originally Class (b) comprised 21 primary tumours tested, and positive for alkaline phosphatase. Four lines failed between passage 3 and passage 11 before retest, leaving the 17 noted as retested. Of these 11 are still in passage, 5 failed to take between the 7th and 28th passage, one was lost at passage 50 through premature death of both recipients. Three of the lines reported in Class (a) have also failed since their test. Nevertheless, most of these lines still persist with passage numbers up to 50 and most are still positive for alkaline phosphatase, although a minority only now produce osteoid.

By selective procedures therefore, one may obtain an osteosarcoma without evident tumour osteoid in violation of the definition of osteosarcoma. We have not yet identified an example of this in the original proband, though in one instance at least the sample of the primary tumour selected for cryostat sections contained so little osteoid that it was missed until the admittedly very osteosarcomatous-looking cells proved positive for alkaline phosphatase.

Perhaps the most intriguing feature of murine osteosarcoma is the common presence of cells positive for acid phosphatase, some of which have morphological features comparable to osteoclasts. According to Owen (1970), among the enzymes of osteoclasts, acid phosphatase

FIG. 12.—Succinic dehydrogenase stain. Osteosarcoma, $^{226}$Ra-induced Ra 41/4, 1st passage; uninucleate and multinucleate cells with positive, black cytoplasm ( × 400).

and succinic dehydrogenase are predominant. The tumours containing cells rich in acid phosphatase, both uni- and multinucleated, also have similar cells rich in succinic dehydrogenase. (Fig. 12). They may be distributed locally or appear diffusely throughout a section. They are often concentrated in a growing edge. The origin of the normal osteoclast is still uncertain. It may be a derivative of an osteoprogenitive cell which can mature to either osteoblast or osteoclast according to demand (Owen, 1970). Alternatively, it has been suggested it is an immigrant scavenger derived perhaps from circulating monocytes (Little, 1973). The distribution in murine osteosarcoma as focal or diffuse peppering is perhaps in favour of an immigrant. However, not all of the acid phosphatase containing cells are multinucleate. These uninucleate cells, the morphology of which is obscured by the colour reaction, may be the mesenchymal precursor cells of osteoclasts (Bingham, Brazell and Owen, 1969) or altered migrant monocytes which fuse to form osteoclasts or undergo endomitosis. Barnes *et al.* (1970) have noted that bone tumours of both osteoblastic and angiosarcomatous nature showed mitoses of normal diploid cells originating from bone marrow as well as of hyperdiploid neoplastic cells derivable from skeletal connective tissue, and chemically induced fibrosarcomata are rich in macrophages (Evans, 1972).

The uninucleate cells positive for acid phosphatase are not specific to osteosarcoma. They were found in 2 of the 3 skeletal tumours negative for alkaline phosphatase and classed as fibrosarcoma of the skeletal connective tissue. They were also found in 4 of 13 sarcomata induced in subcutaneous tissue as reported elsewhere (Barnes *et al.*, 1971). They are compatible with tissue phagocytes (histiocytes). Cells in the spleen, especially the red pulp, and lymph node which could from their distribution be histiocytes give a positive reaction of varying intensity.

In the osteosarcomata, primary or passaged, cells positive for acid phosphatase were less evident in the spindle cell tumours. In the primary tumour there seemed to be no correlation with the amount of tumour-bone present nor with the site and amount of necrosis, but the persistence of bone in passaged tumours seemed to correlate with paucity of acid phosphatase containing cells.

Whereas in some tumours giant cells of the osteoclast form reacting positively for acid phosphatase could be very numerous, we saw no example comparable to the human osteoclastoma or giant cell tumour with stroma negative for alkaline phosphatase (Jeffree and Price, 1965) unless one or both of the two " fibrosarcomata " with acid phosphatase positive giant cells be an example. Giant cells of bizarre form associated with osteosarcomata were negative for acid phosphatase. Both varieties of giant cell could occur in the same osteosarcoma.

*Haemangioendothelial sarcoma.*—The bloody tumours described in B (1) were classified as angiosarcomata (Loutit *et al.* 1973). They are pleomorphic, primitive mesenchymal cell tumours often with atypical vasoformation.

In the normal mouse the endothelia of many small vessels stain positively for alkaline phosphatase. It could be argued that, because the tumours in question are composed of cells which are negative for alkaline phosphatase, they are not endothelial tumours but mimics; but tumours are not composed of mature or necessarily normally functioning cells.

In spite of the rather embryonal appearance of their cells, these tumours are rather indolent of growth, very seldom metastasizing and mortal—it is rare to carry a tumour through 20 passages. A human equivalent of natural occurrence has been described but is rare (Dorfman, Steiner and Jaffe, 1971).

The fully developed tumours are characterized by haemorrhage which may result in much necrosis and therefore a variety of histological appearance. The vasoformative nature of the primary therefore may not always be evident. On passage, however, at early times it is frequently possible to find solid material showing the abnormal capillary vessels. Shortly thereafter formation of small blood cysts occurs, giving an appearance of the tumour to the naked eye, of a blackberry. Later still large cysts containing bloody fluid may form or coagulation may result in an irregular mass of blood, clot and fibrin. In either case passage is effected by material from the outer wall.

It is possible that this group of tumours is not entirely homogeneous and not all of them may be truly haemangioendotheliomatous. Nevertheless, they are a group, distinct from the osteosarcomata, in which there are also differences between tumours and within tumours. Others may have classed them with the less bone productive of osteosarcomata, which may have telangiectatic properties. Indeed sometimes the material available in paraffin embedded sections may be virtually indistinguishable from anaplastic osteosarcoma. It is then that the cryostat section and the negative responses for osteoid in the Taylor's Blue and for alkaline phosphatase become discriminating. Furthermore, on passage the disdinction becomes more marked even to the naked eye. The osteosarcoma, though it may have haemorrhagic patches, is fundamentally a pale tumour especially in early growth; the angiomatous tumour is blood coloured, at all times, and in early growth can be likened in good pathologist's jargon to natural products, in this case, the raspberry or blackberry. Confusion can occur in the special instance, of which we have at least one example (Sr 9/3 H) when both types of tumour tissue occur in the same bone—seen in paraffin section; the cryostat section showed intermixed alkaline phosphatase positive and negative masses of tumour cells. On passage the angiomatous tissue outgrew the osteosarcomatous, so that ultimately a typical angiosarcoma alone remained.

As noted, the angiosarcomatous tumours have been commonly seen in normal mice given strontium-90, the $\beta$ particles of which and of the daughter product, yttrium 90, irradiate bone marrow contained within bone. They have not been seen in mice given radium-226, the $\alpha$ particles of which are much less penetrating. One has been seen after plutonium, which is taken up in bone marrow as well as depositing on bone surfaces (Vaughan, 1973). This one instance occurred in a chimaera, where it could be shown that the tumour arose from host tissue, not the donated bone marrow.

It is likely that these tumours arise predominantly in bone marrow from stromal elements. In mice that have carried strontium-90 for many months the marrow may be haemopoietically aplastic and replaced by a vascular, hyperplastic, connective tissue, in parts of which variation of cell morphology suggests malignant transformation. This could well proceed to the osteolytic type of tumour under discussion. Nilsson (1962) has also described tumour buds of osteosarcoma arising in bone marrow and this is entirely reasonable in that normal marrow contains some stromal cells, other than vascular, which are positive for alkaline phosphatase, perhaps the determinate osteoprogenitive cells (D.O.P.C.) which Friedenstein's experiments indicate exist in bone marrow (Friedenstein *et al.*, 1968).

It is notable that, though one angiosarcoma arose in a chimaera given plutonium, none has been reported in chimaeras given strontium-90. No explanation is offered at this time, but it does indicate another differentiation, and therefore putatively another type of originating cell, from osteosarcoma which is readily induced in chimaeras.

*Fibrosarcoma.*—The three instances described here may be true examples or may be mutated osteosarcomata which have not been diagnosed as such because of inadequate sampling for the presence of tumour bone. Nothing short of serial section would be adequate for complete exclusion. However, fibrosarcoma does arise in many connective tissues, so examples would be expected in bone with its contained bone marrow. Nevertheless the numbers are small compared with those of osteosarcoma and perhaps, because of the paucity in total numbers, there may be no significance in all 3 having been recorded after strontium-90 and none after radium or plutonium. Note that the tumour Ra 49/3 might have been mistaken for a fibrosarcoma but for the evidence of radiograph and paraffin section. On the other hand, whereas mutation from osteoblastoid to fibroblastoid cells seems not uncommon, we have no evidence of a reverse mutation. We have seen instances where alkaline phosphatase positive cells decline nearly to vanishing point, but then in later passages undergo resurgence to an almost dominant population.

We have seen no example of ossifying fibroma of the maxilla, so common in the series reported by Gössner *et al.* (1972) in NMRI mice. The fibrous tissue of these tumours was negative for alkaline phosphatase.

*Lymphoreticular tumours.*—The situation of lymphoreticular tumours with respect to alkaline phosphatase is still unclear, as the following review reveals.

When some of the first tested lymphomata gave positive results, we were not aware of the previous reports going back to Smith (1962) and Metcalf, Sparrow and Wyllie (1962). Haran-Ghera, Hauch-Granoth and Newmann (1972) now suggest that all lymphatic leukaemia cells in SJL/J mice, whether thymic or non-thymic, have raised amounts of alkaline phosphatase determined by a chemical method, whereas cells of granulocytic leukaemias and reticulum cell neoplasms have normal levels.

The localized tumours reported here by the qualitative histochemical method as negative were all tumours of the "reticulum cell" type. The generalized tumours mostly in CBA mice and without gross thymoma were variously positive or negative (weakly positive reactions causing doubt were recorded as negative). In this they were similar to the thymic lymphomata of Doell and Mathieson (1970) in C57BL mice. Within this present series, however, there seemed to be a difference between tumours induced by x-rays, mostly negative and by radionuclides ($^{90}$Sr and Pu) mostly positive. It might be expected that in the case of bone-seeking radionuclides the leukaemic process would be initiated in the irradiated bone marrow and Nilsson (1971) records a focal origin in thoracic vertebrae. In the natural condition and after whole body irradiation the potential sites of induction are more numerous.

There has been some argument as to whether the appearance of alkaline phosphatase in thymic cells is a causal part of the neoplastic process or a consequence. Lagerlöf and Kaplan (1967) used radiation and viruses to induce thymoma in C57BL mice and concluded that development of the reaction was a specific response induced concomitantly with the neoplastic transformation. On the other hand Siegler and Rich (1967) from viral induced thymoma in Swiss mice concluded that neoplastic transformation preceded the appearance of alkaline phosphatase. However, unless there is a complete concordance of neoplasm and positive reaction for alkaline phosphatase the argument, after allowing for the methods used, is inconclusive. The fully developed thymomata of Siegler and Rich were not all histochemically positive, nor were the virally induced tumours of Lumb and Doell (1970), though the chemically induced tumours were. The suggestions (Doell and Mathieson, 1970; Metcalf *et al.*, 1972) that activity for alkaline phosphatase represents chance derepression of an APase gene seems much more acceptable for the thymic lymphomata and the miscellaneous lymphomata investigated by us. However, Haran-Ghera *et al.* (1972) were more impressed by the universal raising of alkaline phosphatase in their SJL/J mice with lymphatic type of leukaemia induced by a variety of means and hinted that chemically induced lymphomata arising from B cells might have higher levels than spontaneous and virally induced neoplasms of T cells (Haran-Ghera and Peled, 1973).

The data of Haran-Ghera *et al.* (1972) are undoubtedly persuasive, but a histologist might regard purely chemical assay as suspect, for alkaline phosphatase is an enzyme widely distributed in tissues and not specific for lymphoma cells.

### CONCLUSIONS

From our experience in staining cryostat sections by the azo-coupling technique for demonstrating alkaline and acid phosphatases and by Taylor's Blue for metachromasia of osteoid we conclude:

(1) True osteosarcomata containing atypical tumour bone and osteoid are like normal osteoblastic cells rich in alkaline phosphatase. Taylor's Blue is a useful aid in finding osteoid when it is scanty. The osteosarcoma tumour cells may all be positive for alkaline phosphatase or some, having undergone mutation, may have lost both this enzyme and the capacity to produce osteoid. This mutation may rarely be seen in the primary tumour. Many tumours, when maintained in compatible hosts by serial passage, show it after a lapse of time. Loss of osteoid precedes loss of alkaline phosphatase. Ultimately by selection in passage the resultant is an anaplastic mesenchymal tumour which by definition can no longer be called osteosarcoma. No histochemical differences have been detected between osteosarcomata induced by the separate radionuclides, $^{90}$Sr, $^{226}$Ra and $^{229}$Pu or, by inference from Gössner *et al.* (1972), $^{222}$Ra.

(2) Osteosarcomata may contain bizarre tumour giant cells or giant cells characteristic of normal osteoclasts or both. The osteoclastic forms only stain for acid phosphatase (and succinic dehydrogenase) and are associated with uninucleate cells having similar histochemical properties so that these may be the precursor cells of osteoclasts. The osteosarcomata containing the cells with acid phosphatase tend to lose their osteoid, but not necessarily their alkaline phosphatase, on serial passage. The tumours which retain their osteogenic capacity over many generations of passage have very few cells staining for acid phosphatase.

(3) Primitive mesenchymal cell tumours often of angiosarcomatous form, having occurred from irradiation of bone marrow from $^{90}$Sr ($^{90}$Y) in encasing bone, or rarely from Pu in the marrow itself, are distinguishable from osteosarcoma by the absence of osteoid and lack of alkaline phosphatase in the tumour cells. They may contain tumour giant cells or occasional cells containing acid phosphatase which are interpreted as tissue phagocytes, but they do not manifest osteoclasts.

(4) Fibrosarcomata of bone encountered rarely and only so far after administration of $^{90}$Sr are distinguishable from osteosarcomata by the absence of osteoid and alkaline phosphatase. They may be former osteosarcomata having mutated very early, as some contain osteoclastic cell forms, or may like the angiosarcomata have arisen in stroma of bone marrow.

(5) Lymphomata have occurred commonly only after administration of $^{90}$Sr and Pu, that is, attributable to irradiation of the bone marrow. The cells of the generalized form may react positively or negatively for alkaline phosphatase—any correlation as yet with B and T type lymphocytes is purely speculative. Lymphomata of the form localized to the abdominal viscera have so far lacked stainable alkaline phosphatase.

We are greatly indebted to Dr C. H. G. Price for technical advice and critical evaluation of much material. We are most grateful to John Asante and Gordon Wilkins for histological and photographic preparations respectively.

### REFERENCES

Barnes, D. W. H., Carr, T. E. F., Evans, E. P. & Loutit, J. F. (1970) $^{90}$Sr-induced Osteosarcomas in Radiation Chimaeras. *Int. J. Radiat. Biol.*, **18**, 531.

Barnes, D. W. H., Evans, E. P. & Loutit, J. F. (1971) Local Origin of Fibroblasts Deduced from Sarcomas Induced in Chimaeras by Implants of Pliable Discs. *Nature, Lond.*, **233**, 267.

Bingham, P. J., Brazell, I. A. & Owen, M. (1969) The Effect of Parathyroid Extract on Cellular Activity and Plasma Calcium Levels *in vivo*. *J. Endocr.*, **45**, 387.

Bland, M. R., Carr, T. E. F., Loutit, J. F. & Sansom, J. M. (1972) Tumours Induced by $^{90}$Sr in Normal and Chimaerical CBA/H Mice. In *Second International Conference on Strontium Metabolism, Glasgow* 1972. CONF-720818 Nat. Tech. Service US Dept. of Commerce. p. 167.

Boocock, G., Danpure, C. J., Popplewell, D. S. & Taylor, D. M. (1970) The Subcellular Distribution of Plutonium in Rat Liver. *Radiat. Res.*, **42**, 381.

Dorfman, H. D., Steiner, G. C. & Jaffe, H. L. (1971) Vascular Tumours of Bone. *Human Path.*, **2**, 349.

Doell, R. G. & Mathieson, B. J. (1970) Absence of Alkaline Phosphatase in Rat Thymic Lymphoma Induced by Murine Sarcoma Virus. *Cancer Res.*, **30**, 2456.

Evans, R. (1972) Macrophages in Syngeneic Animal Tumours. *Transplantation*, **14**, 468.

Finkel, A. J., Miller, C. E. & Hasterlik, R. J. (1969) Radium-induced Malignant Tumors in Man. In *Delayed Effects of Bone-Seeking Radionuclides*, Ed. C. W. Mays, W. S. S. Jee, R. D. Lloyd, B. J. Storer, J. H. Dougherty and G. H. Taylor. Univ. of Utah Press. p. 195.

Friedenstein, A. J., Petrakova, K. V., Kurolesova, A. I. & Frolova, G. P. (1968) Heterotopic Transplants of Bone Marrow. *Transplantation*, **6**, 230.

Gössner, W., Hindringer, B., Luz, A. & Schwabe, M. (1972) Morphologie und Enzymhistochemie $^{224}$Ra-induzierter osteogener Sarcoma bei der maus. *Z. Krebsforsch.*, **68**, 225.

Haran-Ghera, N., Hauch-Granoth, R. & Newmann, H. (1972) Alkaline Phosphatase Activity in Spontaneous and Induced Leukemia in SJL/J Mice. *Cancer Res.*, **32**, 2475.

Haran-Ghera, N. & Peled, A. (1973) Thymus and Bone Marrow Derived Lymphatic Leukaemia in Mice. *Nature, Lond.*, **241**, 396.

International Commission on Radiological Protection (1959) I.C.R.P. Publication 2. *Report of Committee II on Permissible Doses for Internal Radiation.* New York: Pergamon Press.

International Commission on Radiological Protection (1968) I.C.R.P. Publication 11. *A Review of the Radiosensitivity of the Tissues in Bone.* Oxford: Pergamon Press.

Jaffe, H. L. (1958) *Tumors and Tumorous Conditions of the Bones and Joints.* London: Kimpton.

Jeffree, G. M. & Price, C. H. G. (1965) Bone Tumours and their Enzymes. *J. Bone Jt Surg.*, **47B**, 120.

Lagerlöf, B. A. & Kaplan, H. S. (1967) Specificity of the Relationship between Thymus Alkaline Phosphatase Activity and Lymphoma Development in Strain C57BL Mice. *J. natn. Cancer Inst.*, **38**, 437.

Little, K. (1973) *Bone Behaviour.* London: Academic Press. p. 166.

Loutit, J. F., Bland, M. R., Carr, T. E. F., Sansom, J. M. & Smith, C. (1973) Tumours in Bone and Bone Marrow Induced in CBA/H Mice by $^{90}$Sr and $^{226}$Ra. In *Bone—Certain Aspects of Neoplasia.* Colston Papers: no. 24. Ed. C. H. G. Price and F. A. M. Ross. London: Butterworth. pp. 395–408.

Loutit, J. F. & Vaughan, J. M. (1971) The Radiosensitive Tissues in Bone. *Br. J. Radiol.*, **44**, 815.

Lumb, J. R. & Deoll, R. G. (1970) The Biochemical Characterization of Alkaline Phosphatases from Chemical- and Viral-induced Thymic Lymphomas of C57BL Mice. *Cancer Res.*, **30**, 1391.

Metcalf, D., Sparrow, N. & Wyllie, R. (1962) Alkaline Phosphatase Activity in Mouse Lymphoma Tissue. *Aust. J. exp. Biol. med. Sci.*, **40**, 215.

Micklem, H. S. & Loutit, J. F. (1966) *Tissue Grafting and Radiation.* New York: Academic Press. p. 147.

Nilsson, A. (1962) Histogenesis of Sr$^{90}$-induced Osteosarcomas. *Acta vet. scand.*, **3**, 185.

NILSSON, A. (1971) Pathologic Effects of Different Doses of Radiostrontium in Mice. *Acta radiol.*, **10**, 115.

OWEN, M. (1970) The Origin of Bone Cells. *Int. Rev. Cytol.*, **28**, 213.

PRICE, C. H. G. (1958) Primary Bone Forming Tumours and their Relationship to Skeletal Growth. *J. Bone Jt Surg.*, **40B**, 574.

SIEGLER, R. & RICH, M. A. (1967) Significance of Increased Alkaline Phosphatase Activity in Viral-induced Thymic Lymphoma. *Proc. Soc. exp. Biol. Med.*, **125**, 868.

SMITH, C. (1962) Studies on the Thymus of the Mammal. XII Histochemistry of the Thymus of C57BL/6 and AKR Strain Mice. *J. natn. Cancer Inst.*, **26**, 389.—XIII Histochemistry of Irradiated Thymuses of C57BL Strain Mice. *J. natn. Cancer Inst.*, **29**, 375.

TAYLOR, K. B. & JEFFREE, G. M. (1969) A New Basic Metachromatic Dye, 1 : 9 Dimethyl Methylene Blue. *Histochem. J.*, **1**, 199.

VAUGHAN, J. M. (1973) *The Effects of Irradiation of the Skeleton*. Oxford: Clarendon Press. p. 216.